# Finite element biomechanical comparison of bone-driven and occlusion-driven fibular flap positioning in lateral segmental mandibular reconstruction

**DOI:** 10.1016/j.jobcr.2026.101419

**Published:** 2026-02-05

**Authors:** Dmytro Filonenko, Yurii Chepurnyi, Mykola Kryshchuk, Bekir Osmanov, Andrii Kopchak

**Affiliations:** a Department of Maxillofacial Surgery, Bogomolets National Medical University, Kyiv, Ukraine; b Department of Dynamics, Strength of Machines and Materials Resistance, National Technical University of Ukraine “Igor Sikorsky Kyiv Polytechnic Institute”, Kyiv, Ukraine

**Keywords:** Mandibular defect, Mandibular reconstruction, Dental implantation, Microvascular free fibula flap (FFF), Implant-supported prosthesis, Finite-element analysis

## Abstract

**Objective:**

To evaluate, using finite-element analysis (FEA), the stress–strain state of mandibular bone and the bone component of a microvascular free fibula flap (FFF) reconstructing a lateral segmental mandibular defect, comparing two flap positions relevant to implant-supported fixed prosthetic rehabilitation.

**Materials and methods:**

CT-derived three-dimensional models of a reconstructed mandible were generated in two geometries: (A) flap aligned with the inferior mandibular border and (B) flap positioned at the level of the alveolar crest. Each geometry received two endosseous implants and a fixed prosthesis and was subjected to representative masticatory loading (vertical/occlusal and anterior/incisal). Four FEA models (2 positions × 2 load cases) were analyzed, with two predefined regions of interest per model (distal and medial), each examined at the upper and lower margins.

**Results:**

Compared with Model A (inferior mandibular border), Model B (alveolar crest) produced lower peak bone stresses in most analyzed regions (≈10–40% reduction) under mastication-representative static molar and incisal loads. The only exception was the medial–inferior margin under incisal loading, where stress increased markedly (8.96 vs 2.22 MPa; ∼4-fold). Under occlusal loading, medial–inferior stresses were <1 MPa in both models. Across both positioning scenarios, peak crestal bone von Mises stresses clustered near the implant neck (crestal bone); analyses were restricted to bone.

**Conclusion:**

A higher (alveolar-crest) position of the bone component of the FFF yields a more favorable stress–strain environment and load distribution, supporting more favorable biomechanical conditions for osseointegration and long-term function.

## 1 Introduction

Reconstruction of segmental mandibular defects remains a complex challenge in contemporary oral and maxillofacial surgery. Such defects may result from high-energy trauma, ballistic or blast injuries, ablative resections for benign or malignant disease, and radiation- or medication-related osteonecrosis 1, 2, 3. Segmental defects typically involve loss of bone, dentition, and adjacent soft tissues; mucosal and cutaneous deficits are also common. The resulting impairment of mastication, swallowing, speech, and airway function—together with facial deformity—imposes substantial psychological and social burdens and reduces health-related quality of life and overall functional capacity.^4^^,^^5^

The primary goals of management are to restore mandibular continuity, anatomy, and function while providing adequate soft-tissue coverage and minimizing perioperative risk, the number of procedures, and overall rehabilitation time. In addition to osseous and soft-tissue reconstruction, prosthetic rehabilitation—often implant-supported—is a key determinant of long-term treatment success.^6^^,^^7^

Vascularized free fibula flap (FFF) transfer is widely regarded as the most effective option for segmental mandibular reconstruction 8, 9, 10. With a fasciocutaneous or musculocutaneous paddle, it enables simultaneous replacement of extensive osseous defects and associated soft-tissue loss. Implant-supported rehabilitation after FFF reconstruction is well documented, with implant osseointegration rates in transplanted fibula reported to be as high as 98%,^11^ comparable to outcomes in edentulous mandibles.^12^ Nevertheless, a key limitation is the geometric mismatch between the fibula and the native mandibular body, which may complicate—or even preclude—subsequent implant-supported prosthetic rehabilitation.

Several strategies have been described for positioning the FFF bone segment relative to the mandibular remnants, with substantial differences in the feasibility and predictability of implant-supported prosthetic placement. In the bone-driven approach, the FFF is positioned at the mandibular inferior border, providing a close restoration of the native mandibular contour.^13^ However, this configuration increases interarch distance (vertical restorative space) and the crown-to-implant ratio, thereby worsening peri-implant biomechanics and soft-tissue conditions and making prosthesis hygiene more challenging.^14^

In the occlusion-driven approach, the fibular segment is positioned higher, with its superior border approximating the base of the mandibular alveolar process. This improves conditions for subsequent prosthetic rehabilitation but may create a “step” along the inferior mandibular border.^15^ Although these strategies have been discussed extensively from technical and biological perspectives 16, 17, 18, 19, 20, little is known about their biomechanical implications.

Accordingly, each approach has distinct surgical and prosthetic advantages and limitations, while the effect of FFF positioning on stress–strain distribution within bone and around dental implants remains largely unexplored. We hypothesize that these biomechanical parameters are important not only for implant survival but also for overall reconstructive outcomes by influencing bone regeneration and remodeling.

Therefore, the aim of this study was to characterize the stress–strain state of the mandibular bone and transplanted FFF in lateral segmental defects reconstructed with implant-supported prostheses, as a function of FFF segment positioning.

## 2 Methods

### 2.1 Study design and case selection

**Fig. 1:**
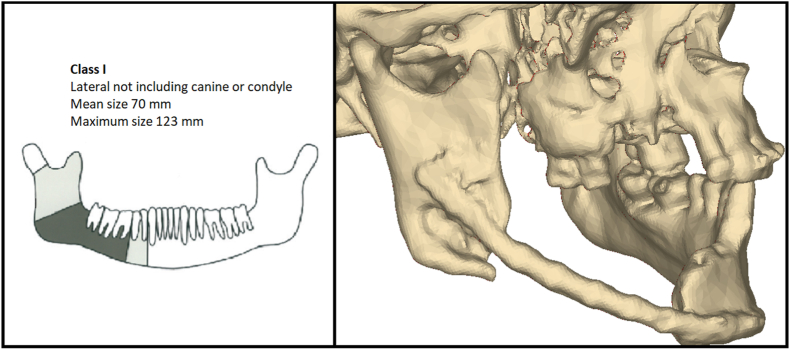
Brown Class I lateral mandibular defect. Left: Schematic. Right: 3D CT reconstruction of the study case showing the lateral segmental defect. (For interpretation of the references to color in this figure legend, the reader is referred to the Web version of this article.)

We constructed and analyzed a series of finite element (FE) models of mandibles with lateral segmental defects reconstructed with FFFs in two positions and restored with fixed implant-supported prostheses. A multislice computed tomography (MSCT) scan of a patient with a lateral mandibular body defect—without involvement of the ramus or canine region (Brown class I^9^)—was used to generate the base geometry (Fig. 1).

### 2.2 Virtual reconstruction and model generation

**Fig. 2:**
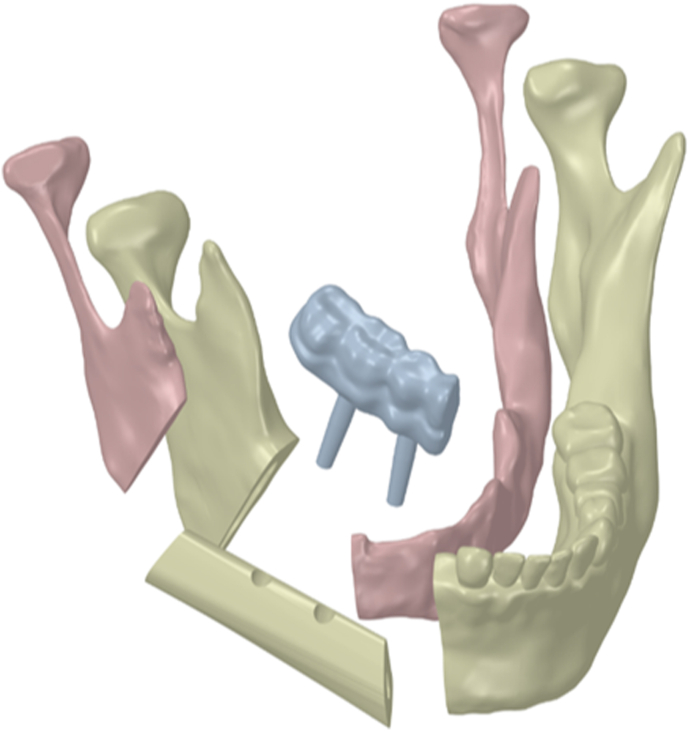
Components of the finite-element model of the reconstructed mandible: cortical and trabecular layers of the preserved mandibular segments; microvascular free fibula flap; two endosseous implants; and the fixed implant-supported prosthesis.

Three-dimensional virtual models with two FFF placement patterns were created in Mimics Medical 25.0 (Materialise, Belgium). Raw DICOM data from the patient's MSCT scans of the facial skeleton and fibula were imported, and segmentation was performed using radiodensity-based thresholds. Separate masks were generated for cortical and trabecular bone in both the mandible and the FFF, and three-dimensional surface meshes were created from each mask. Using software tools and Boolean operations, the segmental mandibular defect was virtually reconstructed with an FFF segment contoured to the required size and shape (Fig. 2).

**Fig. 3:**
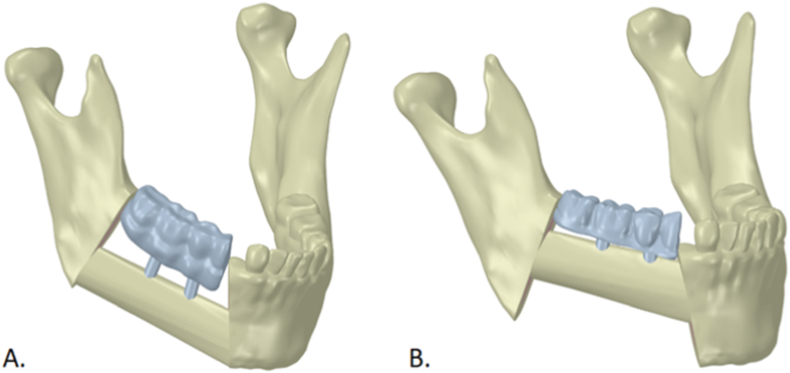
Geometries used for FEA. Model A: fibula flap aligned with the inferior mandibular border (*bone-driven*). Model B: flap positioned at the alveolar crest (*occlusion-driven*). Blue—fixed implant-supported prosthesis; ivory—native mandible and flap. (For interpretation of the references to color in this figure legend, the reader is referred to the Web version of this article.)

Two geometries were then prepared in Ansys (Ansys, Inc., USA) for finite element analysis (FEA): Model A—FFF aligned with the inferior mandibular border; and Model B—FFF positioned at the alveolar crest (Fig. 3).

Three-dimensional models of two standard conical dental implants (10 mm length, 4.0 mm diameter) were created in Autodesk Inventor (Autodesk, Inc., San Rafael, CA, USA) and positioned in prosthetically driven locations within the reconstructed segment 19, 20, 21. A virtual fixed dental prosthesis was subsequently designed in exocad (exocad GmbH, Germany).

### 2.3 Finite-element mesh and material properties

**Fig. 4:**
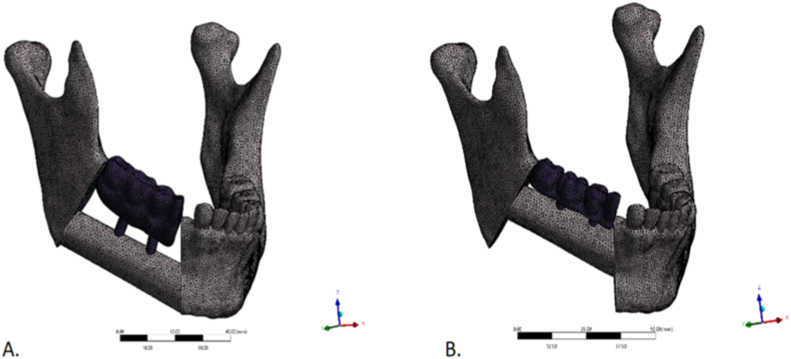
Finite-element meshes of the two reconstructed-mandible geometries. (A): fibula flap aligned with the inferior mandibular border. (B): flap positioned at the alveolar crest. Unstructured tetrahedral meshes with local refinement around the implants and fixed prosthesis.

Preprocessing was performed in Ansys (Ansys, Inc., USA). Separate solid bodies were defined for each component, and unstructured tetrahedral meshes were generated. Linear-elastic material properties were then assigned. Mesh density was determined using a standard mesh-convergence analysis (Fig. 4).

Material properties for bone, titanium implants, and the zirconia framework were obtained from the literature 22, 23, 24. When mandible- or fibula-specific ultimate strength data were unavailable, cortical long-bone reference values were used,^23^ supplemented by published fibular cortical tissue properties.^24^ To simplify the analyses, bone was modeled as a continuous, homogeneous (within each material type), linearly elastic, isotropic material. The elastic modulus was set to 13 GPa for cortical bone and 0.8 GPa for cancellous bone, with Poisson's ratio (ν) of 0.30 for all bone. Implants and the prosthetic framework were assigned standard properties for Ti-6Al-4V (Grade 5) and zirconia ceramic, respectively (E = 114 GPa, ν = 0.34; and E = 180 GPa, ν = 0.25).

### 2.4 Boundary conditions and loading

Loading conditions representative of normal mastication were evaluated. Two load cases were applied to each model: (1) a 100 N vertical load distributed over the occlusal surface of the prosthesis to simulate chewing, and (2) a 100 N vertical load applied at the incisal edge to simulate anterior biting. The preserved mandibular segments were constrained at the temporomandibular joints by fixing the corresponding nodes. These boundary conditions and loading schemes are consistent with prior cranio-maxillofacial FE studies on mandibular fixation and patient-specific constructs.^25^^,^^26^

**Fig. 5:**
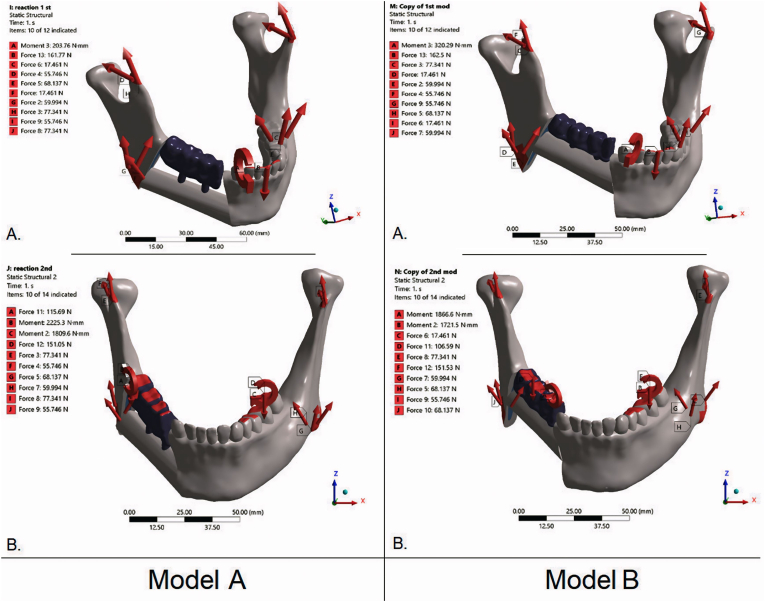
Boundary conditions and load cases for the FEA models. Left: Model A—FFF along the inferior mandibular border (*bone-driven*). Right: Model B—FFF at the alveolar crest (*occlusion-driven*). A: occlusal loading (100 N vertical distributed over the fixed prosthesis). B: incisal loading (100 N vertical applied at the incisal edge). Mandibular segments were constrained at the TMJ regions; red arrows indicate applied loads and reaction forces/moments. (For interpretation of the references to color in this figure legend, the reader is referred to the Web version of this article.)

Contacts between components—including the implants, prosthetic framework, FFF, and cortical and cancellous bone—were modeled as bonded, representing complete bony consolidation and implant osseointegration. In total, four simulations were performed, reflecting two geometries and two loading conditions (Fig. 5).

### 2.5 Outcome measures and data analysis

After solving each model, von Mises (equivalent) stresses were calculated in the transplanted FFF, and stress distributions in the mandible were visualized using color contour maps. Peak equivalent stresses were then compared with literature-based ultimate strength values for cortical and cancellous bone. Reference thresholds used for benchmarking were approximately 90–135 MPa for cortical bone, with lower values for cancellous bone, together with literature-based strength values for the titanium and zirconia components 22, 23, 24^,^^27^.

## 3 Results

**Fig. 6:**
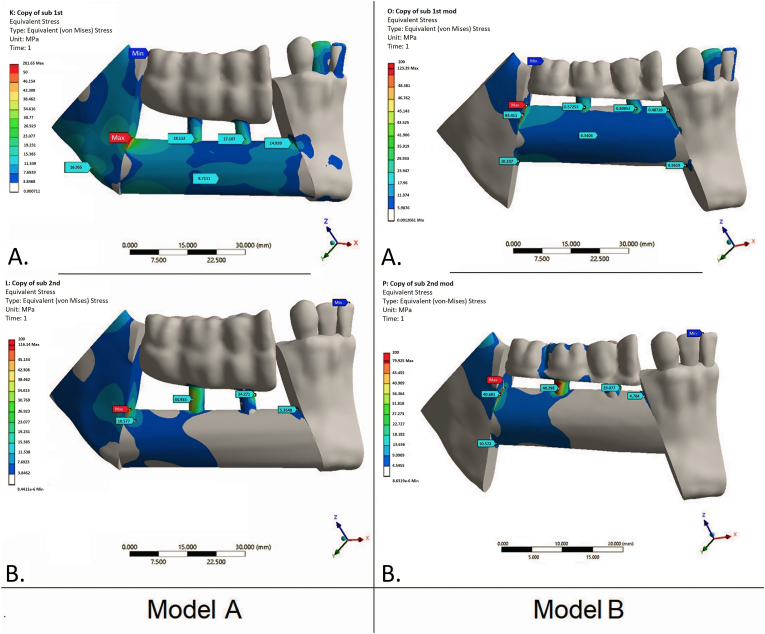
Von Mises equivalent stress maps (MPa) for the two flap positions and load cases. Left column — Model A: FFF aligned with the inferior mandibular border (bone-driven). Right column — Model B: FFF positioned at the level of the alveolar crest (occlusion-driven). A: occlusal loading (100 N vertical distributed over the fixed prosthesis). B: incisal loading (100 N vertical at the incisal edge). Stresses shown for bone only. Peak crestal bone stresses cluster near the implant neck (crestal bone) and along the superior–distal flap margin. Compared with Model A, Model B generally exhibits lower peak bone stresses (≈10–40% across most sites); the notable exception is the medial–lower margin under incisal loading, where stresses are higher in Model B.

Although overall stress–strain patterns and von Mises stress distributions were similar between Model A (inferior FFF) and Model B (superior FFF), peak values differed substantially. Under incisal loading, Model B showed lower peaks at three of four measurement sites (≈10–40% lower than Model A). The only exception was the medial–lower site, where Model B was markedly higher (8.96 vs 2.22 MPa; ∼4-fold). Across both load cases and flap margins, the superior–distal edge of the transplant consistently exhibited the highest stress concentration, likely reflecting the reconstructed mandibular contour (Fig. 6).

Under occlusal loading of the fixed prosthesis, Model B again yielded lower peaks—most prominently at the distal–upper margin (≈39% lower than Model A)—with smaller differences elsewhere (≈7–12%); medial–lower stresses were <1 MPa in both models. Full numerical results are provided in Table 1.

**Table 1:** Peak von Mises equivalent stresses in mandibular bone by fibular flap position and load case.

	Model A (FFF aligned with the inferior mandibular border, *bone-driven*)				Model B (FFF positioned at the level of the alveolar crest, *occlusion-driven*)			
Loading	Incisal loading		Occlusal loading (fixed prosthesis)		Incisal loading		Occlusal loading (fixed prosthesis)	
Margin of the FFF	upper	lower	upper	lower	upper	lower	upper	lower
Maximum stress value in distal part, MPa	201.65	20.34	116.14	11.38	123.39	18.23	70.95	10.57
Maximum stress value in medial part, MPa	14.93	2.22	5.35	<1.00	10.48	8.96	4.70	<1.00

In both positioning scenarios, peak crestal bone stresses were concentrated near the implant neck. Analyses were restricted to bone; stresses within implant components were intentionally deferred to a separate companion study.

In both models, von Mises stresses under the applied loads remained well below the ultimate strength of cortical bone^23^ and did not exceed reported mandibular trabecular thresholds.^22^^,^^27^ However, when extrapolated to peak masticatory forces reported in the literature (600–800 N in the molar region), reconstructed bone in Model A may approach or exceed strength limits.^28^^,^^29^

## 4 Discussion

In the present study, we used CT-based finite element models to compare inferior-border versus alveolar-level fibula free flap positioning after assumed fixation hardware removal and completion of flap consolidation/remodeling, under masticatory loading conditions. Prior studies have applied FEA to evaluate stress–strain distributions in reconstructive plates and fixation systems,^25^^,^^26^ patient-specific endoprostheses,^30^ and bone flaps for mandibular defect reconstruction.^31^ However, many of these studies did not assess biomechanical behavior after dental implant placement and prosthetic restoration. Instead, they primarily addressed risks of non-union and hardware failure in fixation constructs^25^^,^^26^ or early post-reconstruction mechanics of fibula free flaps before dental rehabilitation.^31^

Xian Li et al.^31^ compared FFF placement patterns for lateral mandibular defects and reported that the double-barrel technique is biomechanically advantageous, albeit technically demanding and operator-dependent, with potentially higher surgical risk. In contrast, positioning the flap along the inferior mandibular border resulted in higher stresses and strains at the transplant–mandible interface, particularly under tangential loading.

In our study, peak stresses in the crestal cortical region decreased when the flap was positioned at the alveolar crest level. Stresses in deeper regions remained below critical thresholds in both models, except at the medial–inferior margin under incisal loading, where stresses increased in Model B. Previous studies suggest that crestal cortical bone loss—particularly in the setting of suboptimal prosthesis hygiene and limited keratinized mucosa, which are common after mandibular reconstruction—is associated with peri-implantitis and subsequent implant failure.^32^ Accordingly, reducing stress in the crestal cortical layer may mitigate mechanical overload and help preserve peri-implant bone.

Clinically, occlusion-driven fibular positioning is increasingly relevant in workflows targeting immediate dental rehabilitation, such as the “Jaw in a Day” (JIAD) concept. JIAD represents a contemporary evolution of FFF mandibular reconstruction by integrating virtual surgical planning, immediate implant placement, and delivery of a prefabricated implant-supported provisional prosthesis within a single operative stage. In contrast to conventional bone-driven reconstruction, JIAD is inherently restoration-/occlusion-driven, as the planned prosthetic occlusal plane and implant emergence profiles often dictate the final 3D position of the fibula segments.^33^^,^^34^ This restoratively driven concept may require placing the fibula at an “alveolar-level” position or selecting an orientation in which a specific fibular surface is directed toward the occlusal/prosthetic side to facilitate implant positioning and immediate prosthetic delivery. For example, JIAD workflows may keep the fibula “low” posteriorly along the inferior mandibular border while elevating it anteriorly (symphysis/parasymphysis) to secure approximately 13–18 mm of restorative space, with the lateral surface oriented buccally and implants commonly planned along the anterior (and, when needed, posterior) fibular surface.^35^^,^^36^ Importantly, although immediate (primary) implant placement in JIAD has been hypothesized to jeopardize FFF survival and/or implant osseointegration, comparative studies and meta-analyses show no significant differences in FFF survival^37^^,^^38^ or in implant survival and postoperative infection rates compared with secondary placement.^39^^,^^40^ Within this clinical context, our FE comparison of bone-driven versus occlusion-driven fibular positioning provides a biomechanical perspective on how occlusion-oriented placement—frequently encountered in JIAD planning—may influence stress distribution under functional loading. However, dedicated clinical studies remain necessary to link these biomechanical findings to long-term implant- and prosthesis-related outcomes.

Although the applied loads in our model were comparable to those in an intact mandible and thus may exceed forces typically observed after reconstruction,^28^^,^^29^ the resulting equivalent stresses remained below thresholds expected to induce bone resorption or cortical failure, even in the less favorable implant configuration. Nevertheless, under peak loading conditions, higher stresses could still contribute to bone resorption or structural failure. This should be considered when planning mandibular reconstruction and subsequent prosthetic rehabilitation using vascularized bone flaps.

This study has several strengths. We used a high-resolution, patient-specific CT-derived model of a unilateral lateral segmental mandibular defect reconstructed with a FFF and an implant-supported fixed prosthesis, enabling a clinically realistic yet focused analysis. By keeping flap geometry and implant positions constant, modeling a fully consolidated/osseointegrated reconstruction, and omitting fixation hardware (plates and screws), the simulations isolated the effect of flap positioning on load transfer within the native mandible, fibula segments, and peri-implant bone, while avoiding potential stress shielding and load sharing by osteosynthesis devices.

Several limitations should be acknowledged. First, the analysis was based on a single patient-specific geometry, and bone and reconstructive components were modeled as homogeneous, linearly elastic materials. Second, boundary conditions and loading were simplified (predominantly static vertical forces without explicit masticatory muscle vectors or temporomandibular joint kinematics), and interfaces between bone, flap, and implants were assumed to be perfectly bonded. Third, biological healing and remodeling, as well as material fatigue, were not simulated, and no external (experimental or clinical) validation was performed. Finally, only four simulations (two geometries × two load cases) were analyzed; therefore, inter-individual variability in mandibular shape, cortical thickness, or fibular geometry, as well as alternative flap configurations (e.g., double-barrel or prefabricated constructs), were not assessed.

When coronoid process resection is performed during reconstruction, detachment of jaw elevator muscles—particularly the temporalis—removes a major component of masticatory loading and may alter mandibular biomechanics and stress distribution.^40^ Our FE model does not incorporate these procedure-specific changes in muscle forces; therefore, this represents an additional limitation of the present study.

Accordingly, the reported stress magnitudes and distribution patterns should be interpreted as comparative, hypothesis-generating findings rather than universally generalizable results. Future work should extend these simulations to larger, anatomically diverse cohorts and incorporate more physiologically realistic, time-varying loading conditions, ideally supported by experimental and clinical validation.

## 5 Conclusion

In FE simulations of lateral segmental mandibular reconstruction with a microvascular FFF, osseointegrated implants, and a fixed prosthesis, positioning the flap at the alveolar crest level (Model B) reduced peak bone von Mises stresses compared with positioning along the inferior mandibular border (Model A). Across most analyzed regions, stresses were approximately 10–40% lower in Model B under static molar and incisal loading conditions representative of normal mastication. These findings support occlusion-driven positioning as a biomechanically favorable strategy for implant-supported rehabilitation, while further clinical validation is required.

## Patient consent

This study contains no identifiable patient data or images; individual patient consent is not required.

## Patient's/guardian's consent

No patients were involved in this study. Hence, patient's or guardian's consent was not required.

## Ethical clearance

This research did not involve human participants, identifiable data, or animals; ethics committee approval was not required.

## Ethical clearance

The study protocol was reviewed and approved by the Bioethics and Research Ethics Committee of Bogomolets National Medical University (Protocol No.126, dated February 24, 2025).

## Funding

This research received no external funding.

## Declaration of competing interest

The authors declare that they have no known competing financial interests or personal relationships that could have appeared to influence the work reported in this paper.
